# (18-Crown-6)potassium(I) di­phenyl­stibate(−1)

**DOI:** 10.1107/S1600536814013282

**Published:** 2014-06-14

**Authors:** Marina Kaas, Ute Friedrich, Nikolaus Korber

**Affiliations:** a Institut für Anorganische Chemie, Universität Regensburg, Universitätsstrasse 31, 93053 Regensburg, Germany

## Abstract

Red crystals of the title salt, [K(C_12_H_24_O_6_)][Sb(C_6_H_5_)_2_], were obtained by the reaction of SbPh_3_, KSnBi and 18-crown-6 in liquid ammonia. The asymmetric unit contains one half of a [K(18-crown-6)]^+^ cation and one half of an SbPh_2_^−^ anion, with the central element lying on a twofold axis and a centre of inversion, respectively. In the crystal structure, the sequestered potassium cations show weak inter­actions with the π-electrons of the phenyl groups of the SbPh_2_^−^ anion [shortest K⋯C distances = 3.190 (2) and 3.441 (2) Å], leading to one-dimensional strands along the crystallographic *c* axis. These strands are aligned in a pseudo-hexa­gonal packing perpendicular to the *ab* plane.

## Related literature   

For literature focusing on mechanisms of crystallization and inter­molecular inter­actions or di­phenyl­stibide as a nucleophile, see: Desiraju (2007 ▶); Ugrinov & Sevov (2003 ▶). For a related compound, see Effendy *et al.* (1997 ▶).
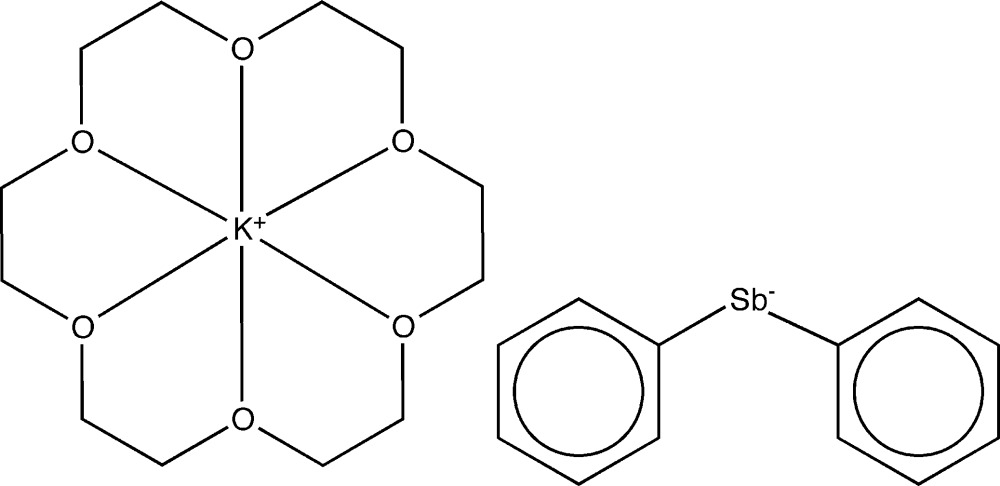


## Experimental   

### 

#### Crystal data  

[K(C_12_H_24_O_6_)][Sb(C_6_H_5_)_2_]
*M* *_r_* = 579.36
Monoclinic, 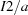

*a* = 15.6933 (9) Å
*b* = 9.2655 (3) Å
*c* = 19.1321 (10) Å
β = 112.654 (6)°
*V* = 2567.3 (2) Å^3^
*Z* = 4
Mo *K*α radiation
μ = 1.27 mm^−1^
*T* = 123 K
0.47 × 0.27 × 0.15 mm

#### Data collection  

Agilent SuperNova (Single source at offset, Eos) diffractometer
Absorption correction: analytical [*CrysAlis PRO* (Agilent, 2012 ▶), using a multi-faceted crystal model based on expressions derived by Clark & Reid (1995 ▶)] *T*_min_ = 0.726, *T*_max_ = 0.863
4194 measured reflections
2592 independent reflections
2297 reflections with *I* > 2σ(*I*)
*R*_int_ = 0.019

#### Refinement  

*R*[*F* ^2^ > 2σ(*F* ^2^)] = 0.023
*wR*(*F* ^2^) = 0.053
*S* = 1.08
2592 reflections
147 parameters
H-atom parameters constrained
Δρ_max_ = 0.39 e Å^−3^
Δρ_min_ = −0.56 e Å^−3^

### 

Data collection: *CrysAlis PRO* (Agilent, 2012 ▶); cell refinement: *CrysAlis PRO*; data reduction: *CrysAlis PRO*; program(s) used to solve structure: *SUPERFLIP* (Palatinus & Chapuis, 2007 ▶); program(s) used to refine structure: *SHELXL97* (Sheldrick, 2008 ▶); molecular graphics: *DIAMOND* (Brandenburg & Putz, 2005 ▶); software used to prepare material for publication: *OLEX2* (Dolomanov *et al.*, 2009 ▶; Bourhis *et al.*, 2011 ▶).

## Supplementary Material

Crystal structure: contains datablock(s) I. DOI: 10.1107/S1600536814013282/ff2129sup1.cif

Structure factors: contains datablock(s) I. DOI: 10.1107/S1600536814013282/ff2129Isup2.hkl

CCDC reference: 1007141

Additional supporting information:  crystallographic information; 3D view; checkCIF report

## Figures and Tables

**Table 1:** Selected bond lengths (Å)

Sb1—C1	2.154 (2)
K1—O2	2.7823 (14)
K1—O3	2.8106 (16)
K1—O1	2.7738 (15)

**Fig. 1.**
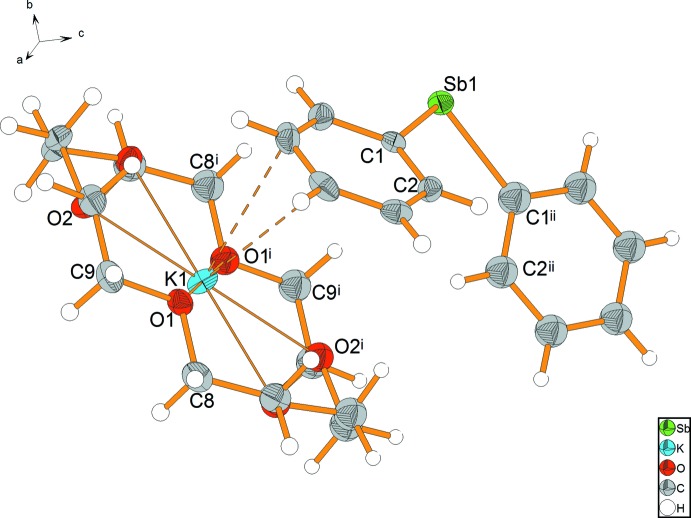
Molecular unit of the title compound with labeling and displacement ellipsoids drawn at the 50% probability level. Symmetry codes: (i) 1.5 - x, 1.5 - y, 1.5 - z, (ii) 1.5 - x, y, 2 - z

**Fig. 2.**
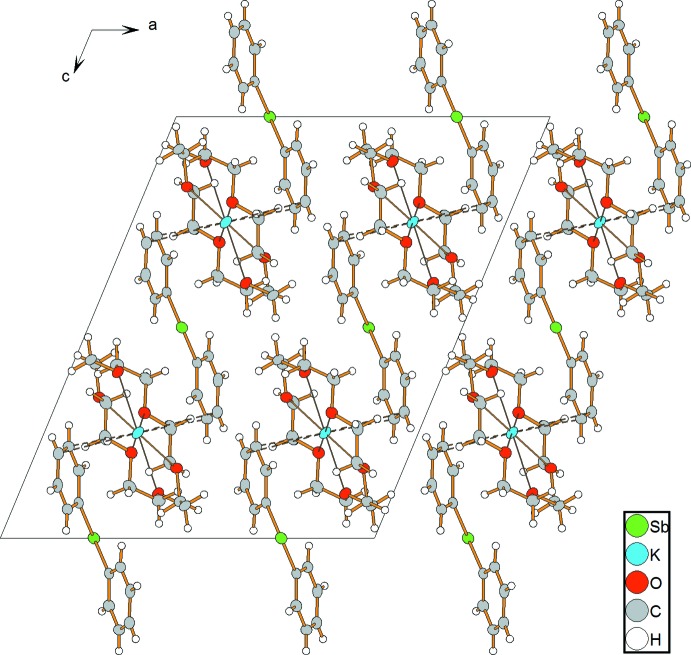
View of the crystal structure showing the one-dimensional strands along the c-axis.

**Fig. 3.**
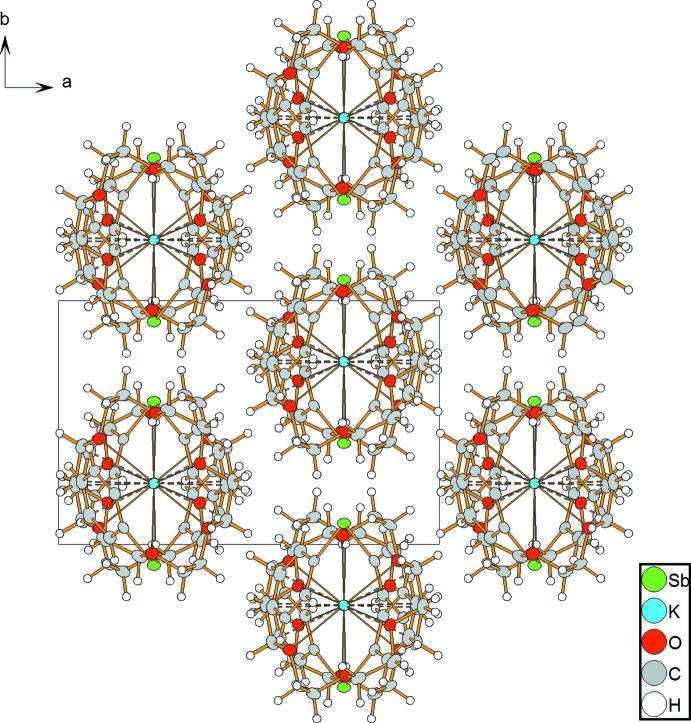
: Projection of the crystal structure showing the pseudo-hexagonal arrangement of the strands perpendicular to the ab-plane.

## supplementary crystallographic information

### S1. Comment

The crystal structure of [K(18-crown-6)]SbPh_2_ was obtained during the investigations of oxidation processes of SnBi-polyanions in liquid ammonia. The asymmetric unit contains one half of a [K(18-crown-6)]^+^-cation together with one half of a SbPh_2_^-^ anion. Figure 1 shows these two molecular ions. K—O bond lengths within the complex range from 2.7738 (15) Å to 2.8106 (15) Å. The Sb—C bond lengths of SbPh_2_^-^-anion of 2.154 (2) Å and the previously reported values for SbPh_3_ of 2.1392 (77) Å - 2.1539 (68) Å (Effendy *et al.*, 1997) are in good agreement. The two phenyl groups of SbPh_2_^-^ exhibit a torsion angle of 28.53 (15)° between C2—C1—Sb1—C1^ii^ and C1—Sb1—C1^ii^—C2^ii^. Short K—C distances suggest π-interactions between the alkali metal cation and the phenyl rings. Two carbon atoms of each phenyl ligand are connected to the cation with a hapticity of two and distances of 3.190 (2) Å and 3.441 (2) Å. Due to symmetry, the SbPh_2_^-^-anion acts as a linker molecule between two [K(18-crown-6)]^+^ complexes that leads to the formation of one dimensional strands along the *c*-axis (Figure 2). The strands are arranged in a hexagonal packing and no further interactions can be found between neighbouring strands (Figure 3).

### S2. Experimental

KSnBi was prepared from the elements in a high temperature synthesis at 723 K in a sealed tube. 88 mg (0.25 mmol) SbPh_3_, 203 mg (0.27 mmol) KSnBi and 66 mg (0.25 mmol) 18-crown-6 were dissolved in dried liquid ammonia in a baked-out reaction vessel. Liquid ammonia was dried over potassium metal and condensed using a standard Schlenk line. The mixture was stored at 237 K for crystallization. Crystals appeared as red blocks in a brownish red solution after 4 weeks.

### S3. Refinement

All H-atoms could be located in the difference map but were positioned with idealized geometry, with *U*iso(H) set to 1.2Ueq of the parent atom. Furthermore there were no irregularities such as dislocation.

### Crystal data

**Table:**

[K(C_12_H_24_O_6_)][Sb(C_6_H_5_)_2_]	*F*(000) = 1184
*M**_r_* = 579.36	*D*_x_ = 1.499 Mg m^−^^3^
Monoclinic, *I*2/*a*	Mo *K*α radiation, λ = 0.71073 Å
*a* = 15.6933 (9) Å	Cell parameters from 1900 reflections
*b* = 9.2655 (3) Å	θ = 3.4–28.0°
*c* = 19.1321 (10) Å	µ = 1.27 mm^−^^1^
β = 112.654 (6)°	*T* = 123 K
*V* = 2567.3 (2) Å^3^	Block, clear reddish red
*Z* = 4	0.47 × 0.27 × 0.15 mm

### Data collection

**Table:**

Agilent SuperNova (Single source at offset, Eos) diffractometer	2297 reflections with *I* > 2σ(*I*)
Graphite monochromator	*R*_int_ = 0.019
phi and ω scans	θ_max_ = 26.4°, θ_min_ = 3.1°
Absorption correction: analytical [*CrysAlis PRO* (Agilent, 2012), using a multi-faceted crystal model based on expressions derived by Clark & Reid (1995)]	*h* = −18→19
*T*_min_ = 0.726, *T*_max_ = 0.863	*k* = −11→10
4194 measured reflections	*l* = −15→23
2592 independent reflections	

### Refinement

**Table:**

Refinement on *F*^2^	Primary atom site location: iterative
Least-squares matrix: full	Hydrogen site location: inferred from neighbouring sites
*R*[*F*^2^ > 2σ(*F*^2^)] = 0.023	H-atom parameters constrained
*wR*(*F*^2^) = 0.053	*w* = 1/[σ^2^(*F*_o_^2^) + (0.019*P*)^2^ + 0.7904*P*] where *P* = (*F*_o_^2^ + 2*F*_c_^2^)/3
*S* = 1.08	(Δ/σ)_max_ = 0.001
2592 reflections	Δρ_max_ = 0.39 e Å^−^^3^
147 parameters	Δρ_min_ = −0.56 e Å^−^^3^
0 restraints	

### Special details

**Table:**

Geometry. All e.s.d.'s (except the e.s.d. in the dihedral angle between two l.s. planes) are estimated using the full covariance matrix. The cell e.s.d.'s are taken into account individually in the estimation of e.s.d.'s in distances, angles and torsion angles; correlations between e.s.d.'s in cell parameters are only used when they are defined by crystal symmetry. An approximate (isotropic) treatment of cell e.s.d.'s is used for estimating e.s.d.'s involving l.s. planes.

### Fractional atomic coordinates and isotropic or equivalent isotropic displacement parameters (Å^2^)

**Table:**

	*x*	*y*	*z*	*U*_iso_*/*U*_eq_	
Sb1	0.7500	1.08580 (2)	1.0000	0.02163 (8)	
K1	0.7500	0.7500	0.7500	0.02190 (15)	
O2	0.62864 (10)	0.83061 (17)	0.60590 (8)	0.0237 (3)	
O3	0.74551 (10)	1.03820 (17)	0.70288 (8)	0.0241 (4)	
O1	0.60734 (10)	0.56706 (17)	0.66609 (8)	0.0225 (3)	
C1	0.67008 (14)	0.9316 (2)	0.91614 (11)	0.0191 (5)	
C10	0.54293 (15)	0.7554 (3)	0.57688 (12)	0.0278 (5)	
H10A	0.5106	0.7774	0.5236	0.033*	
H10B	0.5045	0.7855	0.6036	0.033*	
C4	0.56109 (15)	0.7424 (3)	0.79930 (12)	0.0279 (5)	
H4	0.5263	0.6798	0.7609	0.033*	
C11	0.61898 (16)	0.9803 (3)	0.58922 (12)	0.0292 (5)	
H11A	0.5781	1.0236	0.6106	0.035*	
H11B	0.5924	0.9947	0.5348	0.035*	
C3	0.59436 (14)	0.6990 (3)	0.87465 (12)	0.0249 (5)	
H3	0.5813	0.6068	0.8870	0.030*	
C7	0.66870 (16)	0.3883 (3)	0.76152 (13)	0.0286 (5)	
H7A	0.6253	0.4205	0.7831	0.034*	
H7B	0.6779	0.2853	0.7701	0.034*	
C2	0.64697 (14)	0.7928 (3)	0.93162 (12)	0.0215 (5)	
H2	0.6675	0.7622	0.9817	0.026*	
C5	0.58069 (15)	0.8799 (3)	0.78264 (12)	0.0283 (6)	
H5	0.5577	0.9111	0.7326	0.034*	
C9	0.56103 (15)	0.5973 (3)	0.58705 (12)	0.0256 (5)	
H9A	0.5032	0.5446	0.5668	0.031*	
H9B	0.5991	0.5670	0.5600	0.031*	
C8	0.63067 (16)	0.4186 (3)	0.67829 (13)	0.0267 (5)	
H8A	0.6763	0.3946	0.6574	0.032*	
H8B	0.5762	0.3600	0.6530	0.032*	
C12	0.71193 (16)	1.0495 (3)	0.62224 (12)	0.0276 (5)	
H12A	0.7541	1.0016	0.6037	0.033*	
H12B	0.7075	1.1502	0.6074	0.033*	
C6	0.63440 (15)	0.9725 (3)	0.83964 (12)	0.0248 (5)	
H6	0.6471	1.0645	0.8267	0.030*	

### Atomic displacement parameters (Å^2^)

**Table:**

	*U*^11^	*U*^22^	*U*^33^	*U*^12^	*U*^13^	*U*^23^
Sb1	0.02653 (12)	0.01591 (12)	0.02407 (12)	0.000	0.01153 (9)	0.000
K1	0.0173 (3)	0.0167 (3)	0.0253 (3)	−0.0019 (3)	0.0011 (3)	0.0031 (3)
O2	0.0198 (8)	0.0233 (9)	0.0239 (8)	0.0032 (7)	0.0040 (6)	0.0018 (7)
O3	0.0268 (8)	0.0216 (9)	0.0252 (8)	−0.0006 (7)	0.0114 (7)	0.0043 (7)
O1	0.0253 (8)	0.0221 (9)	0.0194 (7)	−0.0013 (7)	0.0078 (6)	−0.0053 (6)
C1	0.0175 (10)	0.0202 (12)	0.0218 (10)	0.0044 (9)	0.0098 (9)	−0.0001 (9)
C10	0.0185 (11)	0.0411 (16)	0.0187 (11)	0.0017 (11)	0.0016 (9)	−0.0024 (11)
C4	0.0201 (11)	0.0368 (15)	0.0276 (12)	0.0002 (11)	0.0102 (9)	−0.0106 (11)
C11	0.0305 (13)	0.0315 (14)	0.0248 (12)	0.0122 (12)	0.0098 (10)	0.0096 (11)
C3	0.0192 (11)	0.0213 (12)	0.0367 (13)	−0.0001 (10)	0.0134 (10)	−0.0017 (10)
C7	0.0311 (13)	0.0200 (13)	0.0400 (14)	−0.0053 (11)	0.0195 (11)	0.0003 (11)
C2	0.0200 (11)	0.0240 (12)	0.0213 (11)	0.0019 (10)	0.0088 (9)	0.0032 (9)
C5	0.0230 (12)	0.0424 (16)	0.0192 (11)	0.0038 (12)	0.0080 (9)	0.0020 (11)
C9	0.0221 (11)	0.0327 (14)	0.0200 (11)	−0.0061 (11)	0.0058 (9)	−0.0080 (10)
C8	0.0308 (12)	0.0194 (12)	0.0338 (12)	−0.0096 (11)	0.0167 (10)	−0.0076 (10)
C12	0.0356 (13)	0.0236 (13)	0.0286 (12)	0.0084 (11)	0.0178 (10)	0.0090 (10)
C6	0.0245 (12)	0.0264 (13)	0.0251 (11)	0.0020 (11)	0.0114 (9)	0.0041 (10)

### Geometric parameters (Å, º)

**Table:**

Sb1—C1	2.154 (2)	C4—C5	1.376 (3)
K1—O2	2.7823 (14)	C11—H11A	0.9700
K1—O3	2.8106 (16)	C11—H11B	0.9700
K1—O1	2.7738 (15)	C11—C12	1.493 (3)
K1—C4	3.441 (2)	C3—H3	0.9300
K1—C5	3.190 (2)	C3—C2	1.390 (3)
O2—C10	1.424 (3)	C7—H7A	0.9700
O2—C11	1.418 (3)	C7—H7B	0.9700
O3—C7^i^	1.426 (3)	C7—C8	1.496 (3)
O3—C12	1.429 (2)	C2—H2	0.9300
O1—C9	1.431 (2)	C5—H5	0.9300
O1—C8	1.419 (3)	C5—C6	1.389 (3)
C1—C2	1.399 (3)	C9—H9A	0.9700
C1—C6	1.403 (3)	C9—H9B	0.9700
C10—H10A	0.9700	C8—H8A	0.9700
C10—H10B	0.9700	C8—H8B	0.9700
C10—C9	1.490 (3)	C12—H12A	0.9700
C4—H4	0.9300	C12—H12B	0.9700
C4—C3	1.390 (3)	C6—H6	0.9300
			
C1—Sb1—C1^ii^	96.89 (12)	O2—C10—H10A	109.9
O2—K1—O2^i^	180.00 (6)	O2—C10—H10B	109.9
O2^i^—K1—O3^i^	60.27 (4)	O2—C10—C9	109.03 (18)
O2^i^—K1—O3	119.73 (4)	H10A—C10—H10B	108.3
O2—K1—O3^i^	119.73 (4)	C9—C10—H10A	109.9
O2—K1—O3	60.27 (4)	C9—C10—H10B	109.9
O2—K1—C4	86.78 (5)	K1—C4—H4	95.9
O2—K1—C4^i^	93.22 (5)	C3—C4—K1	105.73 (13)
O2^i^—K1—C4	93.22 (5)	C3—C4—H4	120.7
O2^i^—K1—C4^i^	86.78 (5)	C5—C4—K1	67.95 (13)
O2—K1—C5	77.81 (5)	C5—C4—H4	120.7
O2—K1—C5^i^	102.19 (5)	C5—C4—C3	118.7 (2)
O2^i^—K1—C5^i^	77.81 (5)	O2—C11—H11A	109.9
O2^i^—K1—C5	102.19 (5)	O2—C11—H11B	109.9
O3—K1—O3^i^	180.0	O2—C11—C12	108.82 (18)
O3—K1—C4^i^	79.08 (5)	H11A—C11—H11B	108.3
O3^i^—K1—C4^i^	100.92 (5)	C12—C11—H11A	109.9
O3—K1—C4	100.92 (5)	C12—C11—H11B	109.9
O3^i^—K1—C4	79.08 (5)	C4—C3—H3	119.8
O3—K1—C5^i^	102.44 (6)	C2—C3—C4	120.3 (2)
O3^i^—K1—C5^i^	77.56 (6)	C2—C3—H3	119.8
O3^i^—K1—C5	102.44 (6)	O3^i^—C7—H7A	109.8
O3—K1—C5	77.56 (6)	O3^i^—C7—H7B	109.8
O1—K1—O2	59.91 (4)	O3^i^—C7—C8	109.56 (18)
O1—K1—O2^i^	120.09 (5)	H7A—C7—H7B	108.2
O1^i^—K1—O2^i^	59.91 (5)	C8—C7—H7A	109.8
O1^i^—K1—O2	120.09 (5)	C8—C7—H7B	109.8
O1^i^—K1—O3	61.31 (4)	C1—C2—H2	118.9
O1—K1—O3^i^	61.31 (4)	C3—C2—C1	122.2 (2)
O1^i^—K1—O3^i^	118.69 (4)	C3—C2—H2	118.9
O1—K1—O3	118.69 (4)	K1—C5—H5	86.5
O1^i^—K1—O1	180.0	C4—C5—K1	88.49 (14)
O1^i^—K1—C4	116.26 (5)	C4—C5—H5	119.6
O1—K1—C4^i^	116.26 (5)	C4—C5—C6	120.8 (2)
O1—K1—C4	63.74 (5)	C6—C5—K1	95.02 (14)
O1^i^—K1—C4^i^	63.74 (5)	C6—C5—H5	119.6
O1—K1—C5	78.25 (5)	O1—C9—C10	108.91 (18)
O1^i^—K1—C5^i^	78.25 (5)	O1—C9—H9A	109.9
O1^i^—K1—C5	101.75 (5)	O1—C9—H9B	109.9
O1—K1—C5^i^	101.75 (5)	C10—C9—H9A	109.9
C4—K1—C4^i^	180.00 (10)	C10—C9—H9B	109.9
C5—K1—C4	23.57 (6)	H9A—C9—H9B	108.3
C5^i^—K1—C4^i^	23.57 (6)	O1—C8—C7	109.22 (18)
C5—K1—C4^i^	156.43 (6)	O1—C8—H8A	109.8
C5^i^—K1—C4	156.43 (6)	O1—C8—H8B	109.8
C5^i^—K1—C5	180.0	C7—C8—H8A	109.8
C10—O2—K1	115.99 (12)	C7—C8—H8B	109.8
C11—O2—K1	117.29 (12)	H8A—C8—H8B	108.3
C11—O2—C10	112.83 (17)	O3—C12—C11	108.62 (18)
C7^i^—O3—K1	113.46 (12)	O3—C12—H12A	110.0
C7^i^—O3—C12	111.61 (16)	O3—C12—H12B	110.0
C12—O3—K1	111.65 (13)	C11—C12—H12A	110.0
C9—O1—K1	117.40 (12)	C11—C12—H12B	110.0
C8—O1—K1	113.42 (12)	H12A—C12—H12B	108.3
C8—O1—C9	110.95 (17)	C1—C6—H6	119.0
C2—C1—Sb1	125.20 (15)	C5—C6—C1	122.0 (2)
C2—C1—C6	115.9 (2)	C5—C6—H6	119.0
C6—C1—Sb1	118.81 (17)		

Symmetry codes: (i) −*x*+3/2, −*y*+3/2, −*z*+3/2; (ii) −*x*+3/2, *y*, −*z*+2.
